# Morphology-Driven Enhancement of Alkaline OER Performance in Spinel NiCo_2_O_4_ Nanosheet Electrodes

**DOI:** 10.3390/ijms27031444

**Published:** 2026-01-31

**Authors:** Abu Talha Aqueel Ahmed, Abu Saad Ansari, Sangeun Cho, Atanu Jana

**Affiliations:** 1 Division of System Semiconductor, Dongguk University-Seoul, Seoul 04620, Republic of Korea; 2 Nano Center Indonesia Research Institute, Puspiptek Street, South Tangerang 15314, Banten, Indonesia

**Keywords:** oxygen evolution reaction, NiCo_2_O_4_, hydrothermal, intrinsic activity, nanosheets

## Abstract

The oxygen evolution reaction (OER) is a critical anodic process in alkaline water electrolysis, and its catalytic performance can be effectively regulated through rational morphology engineering that governs active-site exposure, mass transport, and charge-transfer kinetics. Herein, we report a precursor-controlled synthesis of spinel NiCo_2_O_4_ (NCO) catalysts with tunable two-dimensional architectures for efficient alkaline OER. By employing hexamethylenetetramine (H) and urea (U) as precipitating agents, the NiCo_2_O_4_ catalysts with distinctly different nanosheet morphologies were directly grown on nickel foam. The NCO-H catalyst exhibits substantially enhanced OER activity by achieving lower overpotential of 259 mV, a smaller Tafel slope of 84 mV dec^−1^, and higher turnover frequency compared to NCO-U catalyst. The superior OER performance is attributed to an ultrathin, highly interconnected nanosheet network that provides abundant accessible active sites, shortened ion-diffusion pathways, and accelerated interfacial charge transfer. Moreover, the optimized electrode demonstrates excellent durability (50 h) with negligible potential degradation after the partial surface transformation into an oxyhydroxide-rich active phase, while post-stability polarization and impedance analyses confirm the preservation of catalytic integrity. These findings highlight precursor-regulated morphology engineering as an effective strategy for optimizing the electrocatalytic performance of spinel oxides and establish NiCo_2_O_4_ as a robust, earth-abundant OER catalyst for alkaline water-splitting applications.

## 1. Introduction

The oxygen evolution reaction (OER) is a crucial anodic process in alkaline water electrolysis, metal–air batteries, and renewable energy conversion systems [1]. However, its practical efficiency is severely limited by sluggish kinetics arising from a multistep four-electron transfer mechanism and complex surface intermediates [2,3,4]. These intrinsic kinetic barriers result in large overpotentials and poor energy efficiency, necessitating the development of highly active, stable, and low-cost electrocatalysts. Although noble metal oxides such as IrO_2_ and RuO_2_ exhibit excellent OER activity [5,6,7,8,9], their scarcity, high cost, and limited durability under alkaline conditions restrict large-scale application [10,11]. Therefore, the earth-abundant transition-metal-based oxides have emerged as promising alternatives to the noble metal-based catalysts [11,12]. Among extensively investigated catalyst materials, the spinel NiCo_2_O_4_ has attracted significant attention owing to its mixed-valence electronic structure, high intrinsic electrical conductivity, and synergistic redox behavior between Ni and Co cations [4,10,13,14]. The coexistence of Ni^2+^/Ni^3+^ and Co^2+^/Co^3+^ redox couples facilitates efficient charge transfer and optimizes the adsorption energy of oxygen-containing intermediates, leading to enhanced OER kinetics [15].

Despite these intrinsic advantages, the OER performance of NiCo_2_O_4_ is highly dependent on its morphology, surface structure, and defect chemistry, which collectively govern the density of exposed active sites, electrolyte diffusion, and charge-transfer resistance [16,17,18]. Morphology engineering has therefore been widely employed to enhance catalytic activity by increasing electrochemically active surface area, shortening ion-diffusion pathways, and improving mass and electron transport [11,18,19,20]. In recent years, various NiCo_2_O_4_ nanostructures, including ultrathin nanosheets, nanowire arrays, hollow nanocages, and hierarchical porous architectures have demonstrated significantly improved OER performance compared with bulk materials [21,22,23,24,25,26]. However, many of these nanostructures are synthesized using template-assisted or surfactant-based methods that involve complicated procedures, limited scalability, and reproducibility issues. In contrast, hydrothermal synthesis using simple precipitating agents offers a facile, scalable, and controllable strategy to tailor nucleation and growth kinetics while maintaining structural tunability.

Based on the critical role of morphology and surface chemistry in governing OER kinetics, this work presents a precursor-controlled hydrothermal route to engineer Co_2_NiO_4_ (NCO) nanosheet architectures with tunable structural features on Ni foam (NF) architecture. By selectively employing urea (U) and hexamethylenetetramine (H) as hydroxide-generating agents, the nucleation and growth behavior was systematically regulated, resulting in distinct nanosheet thicknesses, degrees of interconnection, and surface exposure after thermal conversion to the spinel oxide phase. This synthesis strategy enables precise control over two-dimensional framework formation without the need for templates or surfactants, thereby ensuring scalability and reproducibility. Notably, the optimized interconnected 2D NCO-H nanosheet catalyst electrode exhibits a low OER overpotential of 259 mV at a current density of 10 mA cm^−2^ and even sustained comparatively lower overpotential of 378 mV at 100 mA cm^−2^, outperforming the NCO-U catalyst (305 and 437 mV). Further, the interconnected NCO-H catalyst exhibits a stable and rapid current response at elevated operating current densities and sustained catalytic stability over prolonged electrolysis (50 h). These enhancements are attributed to the resulting ultrathin, highly interconnected nanosheet network provides abundant accessible active sites, shortened ion-diffusion pathways, and improved charge-transfer efficiency, collectively addressing the intrinsic limitations of bulk oxide catalysts. Through this approach, the present study establishes a clear structure-property-activity relationship for Co_2_NiO_4_ and offers practical insights into the rational design of efficient and durable OER electrocatalysts.

## 2. Results and Discussion

### 2.1. Crystallographic Properties of NCO-H and NCO-U Catalyst Electrodes

**Figure 1:**
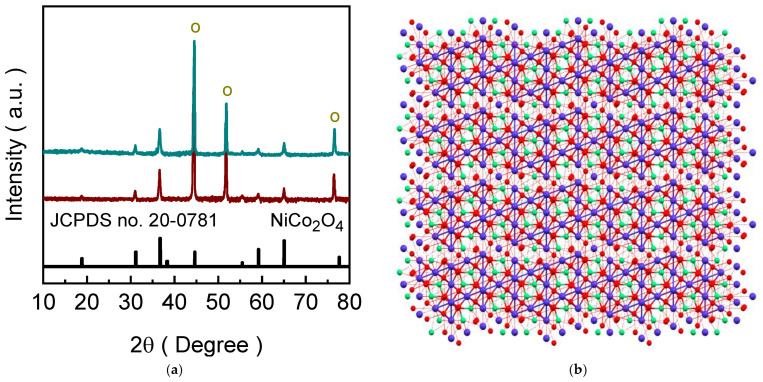
(**a**) XRD patterns of NCO-H (dark cyan) and NCO-U (brown) catalyst electrodes indexed to spinel NiCo_2_O_4_ (JCPDS card number: 20-0781). Notably the intense peaks from the substrate are marked with circles. (**b**) Schematic of the cubic spinel NiCo_2_O_4_ lattice showing Ni/Co cation distribution in tetrahedral and octahedral coordination within the oxygen framework. Ni, Co, and O atoms in NiCo_2_O_4_ are shown in green, purple, and red colors.

The material phase identification and crystallographic arrangement of the prepared NCO-H and NCO-U catalyst electrodes were examined through X-ray diffraction (XRD) technique. Figure 1a shows the X-ray diffraction (XRD) patterns of NCO-H and NCO-U catalyst electrodes. Three intense reflections marked with circle (o) appearing at 44.46°, 51.84°, and 76.56° are attributed to the underlying nickel foam substrate, corresponding to the (111), (200), and (220) facets of face-centered cubic Ni, respectively. All the diffraction peaks can be well indexed to the standard cubic spinel NiCo_2_O_4_ phase (JCPDS card number: 20-0781), confirming the formation of a single-phase cubic structure in both catalyst electrodes. The characteristic reflections corresponding to the (111), (220), (311), (400), (422), (511), and (440) planes are clearly observed at the 2θ spectral angle of 18.86, 31.05°, 36.63°, 55.47°, 59.19°, and 65.05°, indicating the good crystallinity and long-range structural order [27]. No additional impurity-related peaks are detected, suggesting high phase purity irrespective of the chelating agent used during synthesis. Minor variations in the peak intensity and broadening for NCO-H catalyst electrode arise from differences in crystallite size, and microstrain compared to the NCO-U electrode, while the overall cubic symmetry remains unchanged. Figure 1b depicts the crystal structure model of cubic NiCo_2_O_4_ (mp-1096547), illustrating the ordered spinel framework. In this structure, Ni^2+^ ions predominantly occupy octahedral sites, while Co^3+^/Co^2+^ ions are distributed between octahedral and tetrahedral positions, coordinated by oxygen atoms to form a three-dimensional network of edge- and corner-sharing polyhedral arrangement. The periodic arrangement of cations and oxygen anions results in a highly symmetric cubic lattice, consistent with the XRD analysis. This robust and interconnected structure facilitates efficient charge transport and structural stability, which are essential for electrochemical and catalytic applications.

### 2.2. Morphological and Compositional Properties of NCO-H and NCO-H Catalyst Electrodes

**Figure 2:**
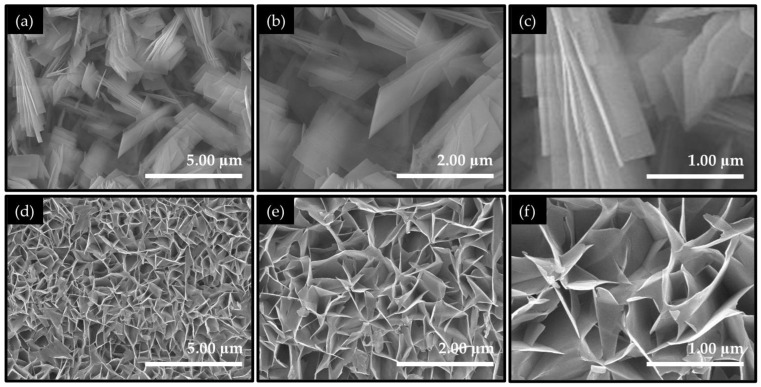
FE-SEM images of NiCo_2_O_4_ catalyst electrodes prepared from different precursors: (**a**–**c**) NCO-U and (**d**–**f**) NCO-H recorded at low to high magnifications.

The FE-SEM images (Figure 2a–f) reveal that the choice of precipitating agent plays a decisive role in directing the morphological evolution of the NiCo_2_O_4_ catalyst electrodes. For the NCO-U catalyst electrode (Figure 2a–c), the electrode surface is composed of compactly arranged, elongated two-dimensional (2D) plate-like nanosheets that collectively form a dense lamellar assembly dominated by a dense assembly. These nanosheets overlap extensively and are tightly stacked, generating a relatively smooth and continuous surface coverage across the nickel foam scaffold. High-resolution images (Figure 2b,c) show that the nanosheets possess well-defined planar faces and sharp edges, with minimal interlayer spacing, indicating a growth mode governed by slow and regulated nucleation. Such morphology is consistent with the gradual hydrolysis behavior of urea, which releases hydroxide ions in a controlled manner, allowing sustained crystal growth and favoring the formation of closely packed two-dimensional structures. Whereas the NCO-H catalyst electrode (Figure 2d–f) exhibits a substantially more open and textured architecture. Instead of compact stacking, the surface is dominated by a highly porous nanosheet network with extensive interconnections throughout the electrode framework. At the higher magnifications (Figure 2e,f), the nanosheets appear significantly thinner, corrugated, and loosely intertwined, forming an interconnected matrix with abundant voids and channels. This hierarchical arrangement introduces a large number of exposed edges, junctions, and open pathways, resulting in a markedly increased surface roughness and accessible catalytically active surface area (*ECSA*). The formation of this porous nanosheet network can be attributed to the rapid decomposition of hexamethylenetetramine, which accelerates nucleation and promotes dynamic self-assembly, thereby suppressing the dense stacking and enabling the development of an open and interconnected 2D nanosheet morphology. Therefore, the comparative FE-SEM analysis highlights that precursor-controlled synthesis effectively tunes the nanosheet organization from a compact lamellar configuration to a highly interconnected network, which is expected to significantly influence electrolyte accessibility, active-site exposure, and charge transport behavior during electrochemical operation.

The FE-SEM-EDS analyses of NCO-H and NCO-U confirm the successful formation and high compositional purity of the spinel NiCo_2_O_4_ phase (Figure S1). In both spectra, only O, Co, and Ni signals are observed, with no detectable impurity-related peaks, indicating clean synthesis. The intense oxygen peak at low energy originates from the oxide lattice, while the characteristic Co and Ni Kα/Kβ peaks appearing in the ~6–9 keV range verify the incorporation of both transition metals into the spinel framework. The quantitative EDS results show atomic percentages of Co (28.47%), Ni (14.11%), and O (57.42%) for the interconnected 2D NCO-H nanosheet network and Co (29.23%), Ni (13.91%), and O (56.86%) for the NCO-U nanosheet electrode, which are reasonably consistent with the theoretical stoichiometry of NiCo_2_O_4_, considering the semi-quantitative and surface-sensitive nature of EDS measurements. The slightly higher oxygen content in both samples is typical for metal oxides and may also include contributions from surface-adsorbed or defect-related oxygen species.

### 2.3. Chemical Bonding States of NCO-H Catalyst Electrodes

**Figure 3:**
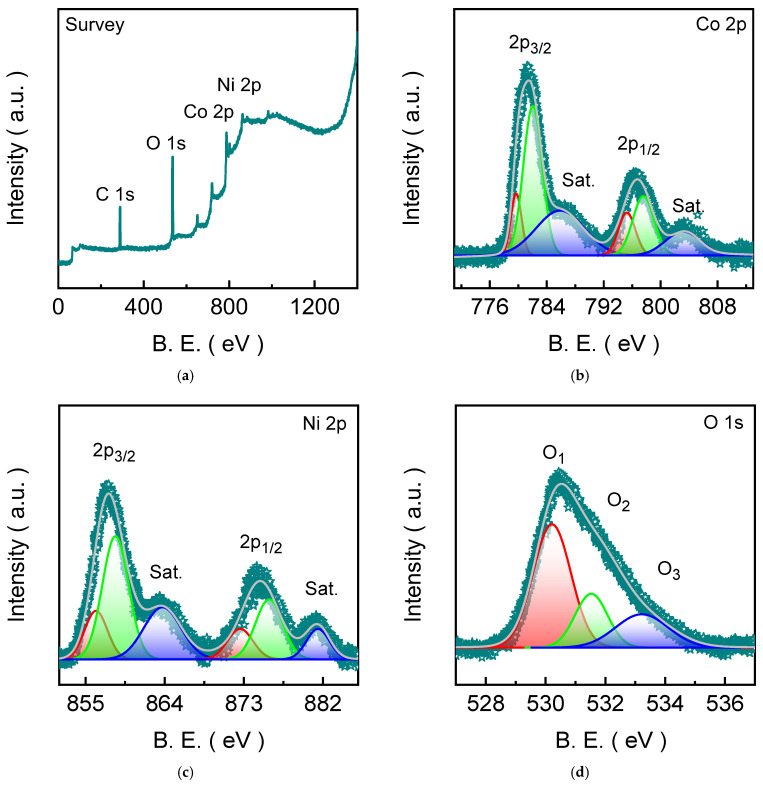
XPS characterization of the NCO-H catalyst electrode: (**a**) survey spectrum showing the presence of Co, Ni, and O, high-resolution deconvoluted emission spectra of (**b**) Ni 2p, (**c**) Co 2p, and (**d**) O 1s core levels. Notably, dark cyan represents the measured XPS spectrum, whereas the colors, such as, light grey, red, green, and blue represent the Gaussian fitted XPS curves.

The X-ray photoelectron spectroscopy (XPS) was employed to elucidate the surface chemical composition and electronic structure of the NCO-H catalyst electrode. The wide-scan spectrum (Figure 3a) confirms the presence of Ni, Co, O, C, and N elements without any extraneous impurity signals, reflecting the high surface cleanliness of the material. The clearly resolved Ni 2p and Co 2p photoelectron features verify the successful incorporation of both transition metals within the spinel NiCo_2_O_4_ lattice, while the intense O 1s signal originates from lattice oxygen and surface-associated oxygen species. The binding energy positions of these core levels are consistent with those reported for spinel-type mixed metal oxides, confirming the preservation of the NiCo_2_O_4_ framework [28,29]. The additional detectable C 1s (285.92 eV) aroused from the contaminant carbon [15,30]. The high-resolution Co 2p spectrum (Figure 3b) reveals two dominant spin-orbit doublets assigned to Co 2p_3/2_ and Co 2p_1/2_, accompanied by distinct satellite features at higher binding energies. The asymmetric nature of the main peaks necessitates the multiple component fitting, indicating the coexistence of Co^2+^ (782.03 and 797.62 eV) and Co^3+^ (779.74 and 795.21 eV) species at the surface [11]. The presence of well-defined shake-up satellites (785.87 and 803.11 eV) is an indicative of mixed Co^2+^/Co^3+^ states of cobalt in the spinel structure with strong metal-oxygen hybridization [29,31]. This mixed cobalt valence environment is a defining characteristic of spinel oxides and plays a key role in facilitating the reversible redox transitions and efficient charge transport during electrochemical reactions.

Further the quantitative insights are obtained from the Ni 2p spectrum (Figure 3c), which exhibits characteristic Ni 2p_3/2_ and Ni 2p_1/2_ peaks along with prominent satellite (Sat. situated at 863.76 and 881.43 eV) structures. The deconvolution peaks confirm the simultaneous presence of Ni^2+^ (856.18 and 872.62 eV) and Ni^3+^ (858.43 and 875.82 eV) species, reflecting a dynamic electronic configuration within the spinel lattice [29]. The pronounced satellite peaks highlight strong Ni-O covalency and charge-transfer interactions, which are known to promote electronic conductivity and redox flexibility in nickel-based oxides. The coexistence of multiple nickel oxidation states suggests an electronically adaptive surface capable of accommodating charge redistribution under electrochemical polarization. The O 1s spectrum (Figure 3d) further clarifies the surface oxygen chemistry. The dominant low-binding-energy component corresponds to lattice oxygen (O_1_) bonded to Ni and Co within the spinel framework, confirming structural robustness. A secondary contribution at intermediate binding energy (O_2_) is associated with oxygen vacancy species, which are often linked to enhanced catalytic activity through improved adsorption characteristics [32]. The higher-binding-energy peak (O_3_) arises from hydroxyl-related environments or chemisorbed water at the surface [33]. All these features indicate a chemically heterogeneous surface with abundant reactive oxygen environments. Nevertheless, the XPS results demonstrate that NCO-H possesses a mixed-valence Ni-Co spinel backbone combined with an oxygen vacancy-related defect. The combination of multivalent metal centers and surface-modulated oxygen chemistry provides a favorable electronic landscape for rapid charge transfer and enhanced electrochemical OER performance.

### 2.4. Electrochemical OER Performances of NCO-H and NCO-U Catalyst Electrodes

The electrocatalytic OER performance of the NCO-H and NCO-U catalyst electrodes were systematically evaluated in 1.0 M KOH using linear sweep voltammetry (LSV) at a scan rate of 1.0 mV s^−1^. All electrochemical potentials initially recorded versus the saturated calomel electrode (SCE) were then converted to the reversible hydrogen electrode (RHE) scale using the following relation:

*E*_RHE_ = *E*_SCE_° + (0.059 × *pH*) + *E*_SCE_, (1)

where *E*_SCE_ is the experimentally measured potential, *E*_SCE_° (0.241 V) is the standard potential of SCE at room temperature, and the pH is the hydronium ion concentration for 1.0 M KOH electrolyte. In addition, the ohmic losses arising from electrolyte resistance were corrected according to the following equation:

*E*_RHE_ (*iR*-corrected) = *E*_RHE_ − (*R*s × *i*), (2)

**Figure 4:**
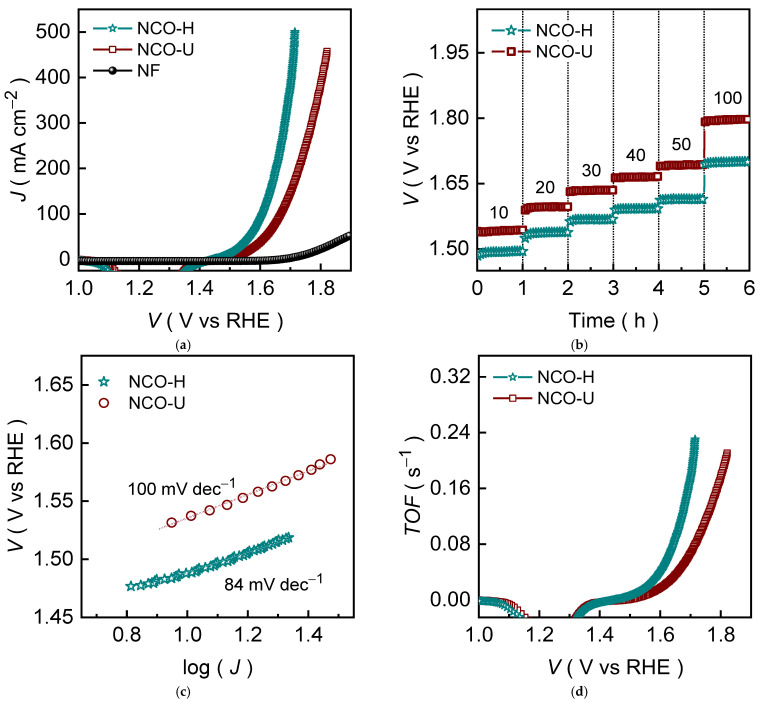
(**a**) LSV curves, (**b**) Voltage-step profile, (**c**) Tafel slopes, and (**d**) TOF plots for the NCO-H and NCO-U nanosheet catalysts.

*η* = *E*_RHE_ (*iR*-corrected) − 1.23, (3)

where *i* is the applied current density (*J*) and *R*_S_ represents the solution resistance obtained from high-frequency intercepts of electrochemical impedance spectra (EIS). Figure 4a presents the *iR*-corrected polarization curves for NCO-H, bare NF, and NCO-U catalyst electrodes. The Ni foam substrate exhibits insignificant anodic current throughout the examined potential window, confirming that the observed OER activity originates exclusively from the deposited catalyst materials. The 2D interconnected NCO-U and NCO-H nanosheet catalysts display substantially enhanced OER responses compared with the substrate, highlighting the intrinsic catalytic contribution of the NiCo_2_O_4_ oxide phase. Notably, the 2D interconnected NCO-H nanosheet catalyst delivers a significantly lower overpotential of 259 mV to achieve a benchmark current density of 10 mA cm^−2^ compared with NCO-U nanosheet (305 mV) and various Ni-/Co-based OER catalyst (Table S1), indicating the superior catalytic efficiency of NCO-H catalyst for OER. As the current density is progressively increased, the potential gap between the two catalysts becomes more pronounced. At the higher current densities of 25, 50, 100, and 250 mA cm^−2^ the 2D interconnected NCO-H nanosheet catalyst achieves the smaller overpotentials of 296, 334, 378, and 441 mV. Whereas the NCO-U nanosheet catalyst consistently maintains a cooperatively higher anodic potential (347, 288, 437, and 519 mV), reflecting more favorable reaction kinetics and improved charge-transfer characteristics for NCO-H catalyst (Figure S2 and Table S2). The enhanced OER activity of NCO-H catalyst can be directly correlated with its interconnected ultrathin nanosheet architecture, which facilitates rapid electrolyte penetration (Figure S2) and provides a high density of exposed active sites (Figure S3). The reduced nanosheet thickness and open framework shorten ion diffusion pathways and promote efficient removal of evolved oxygen bubbles, thereby mitigating local mass-transport limitations.

The operational robustness and potential response of the NCO-H and NCO-U nanosheet catalysts under the diverse working conditions were further assessed using stepwise chronopotentiometric measurements. The applied anodic current density was sequentially increased from 10 to 50 mA cm^−2^ in increments of 10 mA cm^−2^ followed by a final hike of 50 mA cm^−2^ to reach 100 mA cm^−2^. Each current step was maintained for about an hour to allow the electrode potential to reach a steady-state value before proceeding to the next current level. As shown in Figure 4b, NCO-H and NCO-U nanosheet catalysts exhibit distinct and well-defined potential plateaus at each applied current density, indicating stable electrochemical performance and rapid potential stabilization without noticeable voltage fluctuation during the examined period, suggests strong tolerance toward high-current operation and mitigated mass-transport limitations. Further, the 2D interconnected NCO-H nanosheet catalyst steadily showcases a lower operating potential than NCO-U nanosheet catalyst throughout the applied current density range, which further supports the superior OER performance of the NCO-H than NCO-U catalyst obtained from the polarization curves. The superior voltage response of NCO-H can be attributed to its interconnected ultrathin nanosheet architecture, which offers a larger electrochemically accessible surface area and continuous electron transport pathways. Further, interconnected nanosheet structural configuration promotes uniform current distribution and efficient oxygen bubble release, even under elevated current densities. The open and porous network facilitates rapid electrolyte penetration and minimizes local current crowding, enabling uniform reaction distribution even under elevated current densities.

To further clarify the intrinsic OER kinetics of the two electrodes, the Tafel plots were derived from the *iR*-corrected polarization curves (Figure 4c) according to the Tafel relationship as follows [34,35,36,37]:

*η* = α + (log(i) × *β*), (4)

where α is the intercept related to exchange current behavior and *β* is the Tafel slope, which reflects the kinetic barrier of the OER process. The 2D interconnected NCO-H nanosheet catalyst exhibits a markedly smaller Tafel slope of 84 mV dec^−1^ (Figure 4c) compared with the NCO-U (100 mV dec^−1^), indicating more favorable reaction kinetics and faster charge-transfer behavior on the interconnected nanosheet network, which is evidence by *ECSA* compensated LSV curve (Figure S4a). A reduced Tafel slope implies that a smaller potential increment is required, which directly renders to improved catalytic efficiency under various operating conditions. This kinetic advantage is consistent with the LSV results and supports the superior OER activity of NCO-H across both low and high current density regimes. The improved kinetics of NCO-H can be attributed to its interconnected ultrathin nanosheet network, which facilitates higher active-site accessibility, improved electrolyte penetration, and reduced charge-transfer resistance. Thus, the Tafel analysis confirms that precursor-controlled synthesis not only tunes the morphological structure but also improves intrinsic OER kinetics, reinforcing the structure-activity correlation observed throughout this study.

**Figure 5:**
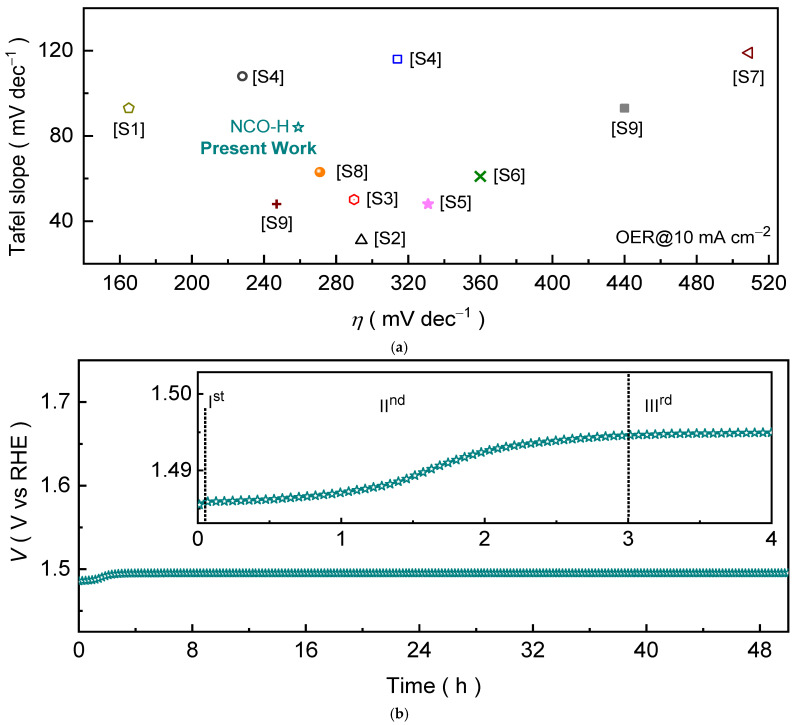
(**a**) Overview of reported OER performance from the literature. (**b**) Chronopotentiometric stability evaluation conducted at current densities of 10 cm^−2^ over 50 h of continuous operation. Inset: magnified view of the initial stability region, highlighting the potential evolution during the early stage of operation.

The kinetic advantages inferred from the Tafel slope results were further substantiate through the turnover frequency (*TOF*) analysis. As the *TOF* reflects the number of oxygen molecules generated per active site per second and therefore provides a more meaningful comparison of intrinsic activity than geometric current density alone. The TOF values were estimated from the measured current using the relation [38,39]:

*TOF* = ((*J* × A)/(4 × *n* × *F*)), (5)

where *A* is the geometric area of the electrode (cm^2^), *F* is the Faraday constant (96,485 C mol^−1^), and the factor 4 accounts for the four-electron transfer process in OER [32]. The term *n* (=*M*/*MW*) represents the molar amount of active catalyst loaded on the electrode and was estimated from the catalyst mass loading (*M*) and molecular weight (*MW*), assuming full participation of the deposited NiCo_2_O_4_ in the OER. The 2D interconnected NCO-H nanosheet catalyst exhibits consistently higher *TOF* values (Figure 4d) than NCO-U catalyst over the investigated potential range, indicating a higher intrinsic turnover rate of OER at comparable driving potentials. This observation agrees well with the smaller Tafel slope of NCO-H and confirms that its enhanced performance is not solely due to higher surface area but also arises from improved site efficiency and accelerated reaction kinetics. The higher TOF of NCO-H can be attributed to its ultrathin, interconnected nanosheet architecture, which improves active-site exposure and facilitates faster charge transfer, as supported by the EIS results. Furthermore, the improved performance of NCO-H is quite reliable (Figure S5) and comparable to many reported non-noble Ni-/Co-based oxide catalysts under similar alkaline conditions (Figure 5a and Table S1).

Thereafter, the long-term operational stability of the interconnected 2D NCO-H nanosheet catalyst was evaluated by chronopotentiometry at a constant anodic current density in an alkaline 1.0 M KOH for 50 h. The catalyst maintains a largely stable potential profile throughout the test, indicating robust durability under continuous oxygen evolution conditions. Notably, a slight potential variation is observed during the initial stage of electrolysis (inset), followed by stabilization to an almost constant value over prolonged operation. This initial potential change in the chronopotentiometric test is commonly associated with electrochemical surface reconstruction, where the as-synthesized spinel oxide undergoes partial oxidation and transforms at the near-surface region into an oxyhydroxide-rich layer under alkaline anodic polarization driven by the electrooxidation process. The increase in Co^3+^/Ni^3+^ contribution together with the enhanced oxyhydroxide-related O 1s component observed in post-OER XPS (Figure S4b–d) indicates electrochemically induced surface reconstruction toward a CoOOH-like active phase. This process leads to the Co/Ni cations shifting toward higher valence states, which are widely considered the true catalytically active phases in alkaline OER. While both Co and Ni participate in the redox evolution, the Co^2+^/Co^3+^ transition is generally considered more directly involved in OER kinetics through the generation of CoOOH, whereas Ni^2+^/Ni^3+^ species mainly contribute by improving electronic conductivity and stabilizing the reconstructed surface [40,41,42,43,44,45,46]. The formation of this oxyhydroxide-rich layer modifies the interfacial resistance and adsorption behavior of reaction intermediates, accounting for the initial potential variation observed in the inset (Figure 5b). After this activation stage, the potential becomes steady, suggesting that a stable and fully reconstructed active surface has formed and that the catalyst maintains its structural integrity without noticeable degradation. The persistence of a constant operating potential further confirms the electrochemical robustness of the reconstructed surface during prolonged OER operation. Nonetheless, the negligible potential drift observed after the initial activation period confirms the excellent structural and electrochemical robustness of the catalyst under sustained OER conditions. Importantly, this outstanding durability is further corroborated by post-stability electrochemical measurements, where the Nyquist impedance spectrum (Figure S6a) and the polarization curves (Figure S6b) recorded after the 50 h stability test exhibit minimal deviation from their initial profiles. The nearly unchanged overpotential and charge-transfer resistance indicate that the active surface and electronic pathways remain well preserved during prolonged operation. These results collectively demonstrate that the catalyst not only undergoes a favorable surface reconstruction into an oxyhydroxide-rich active phase but also maintains long-term operational integrity, making it highly suitable for practical alkaline OER applications.

## 3. Materials and Methods

### 3.1. Materials

All chemicals were of analytical grade and used without further purification obtained from a commercial supplier (Sigma-Aldrich, St. Louis, MO, USA). Ethanol (CH_3_CH_2_OH, ≥99.5%), hydrochloric acid (HCl, 37%), cobalt nitrate hexahydrate (Co(NO_3_)_2_·6H_2_O, ≥99%), acetone (CH_3_COCH_3_, ≥99.5%), potassium hydroxide (KOH, ≥85%), hexamethylenetetramine (C_6_H_12_N_4_, ≥99%), Nickel nitrate hexahydrate (Ni(NO_3_)_2_·6H_2_O, ≥99%), ammonium fluoride (NH_4_F, ≥98%), and urea (U, CO(NH_2_)_2_, ≥99%). Deionized water (DIW) was used in all experimental procedures. Three-dimensional (3D) microporous NF was obtained from Alantum (Seoul, Republic of Korea). Prior to use as conductive substrate, the NF was cut into pieces of 1 × 5 cm^2^ and sequentially cleaned by ultrasonication in dilute HCl, ethanol, acetone, and DIW for 10 min (min.) each to remove surface oxides followed by thorough rinsing with ethanol and DIW. The cleaned substrates were finally dried under overnight vacuum furnace before use.

### 3.2. Synthesis of NCO-H and NCO-U Catalyst Electrodes

The NiCo_2_O_4_ catalyst electrodes were directly grown on nickel foam via a simple hydrothermal route. In a typical synthesis, stoichiometric amounts of Co(NO_3_)_2_·6H_2_O and Ni(NO_3_)_2_·6H_2_O were dissolved in DIW to obtain a clear precursor solution with a Co:Ni molar ratio of 2:1. A hexamethylenetetramine (36 mmol) and NH_4_F (12 mmol) were subsequently introduced under continuous stirring at room temperature. The pretreated NF substrate was immersed in the resulting solution, sealed in a Teflon-lined autoclave, and heated for 6 h at 120 °C. After naturally cooling, the deposited catalyst electrodes were rinsed thoroughly with ethanol and DIW, dried overnight, and annealed in air at 350 °C for 3 h to yield the NiCo_2_O_4_ (NCO-H) electrode. For comparison, the urea assisted NiCo_2_O_4_ (NCO-U) catalyst electrode was then prepared under identical conditions by replacing hexamethylenetetramine with urea as the base source. The morphology and phase evolution of NiCo_2_O_4_ catalyst electrodes grown on NF are strongly governed by the nature of the base source employed during hydrothermal synthesis. The hexamethylenetetramine hydrolyses through C_6_H_12_N_4_ + 6H_2_O → 6HCHO + 4NH_3_ and subsequent NH_3_ + H_2_O ⇋ NH_4_^+^ + OH^−^, resulting in a gradual and homogeneous release of OH^−^ ions with minimal gas evolution. This progressive increase in local alkalinity promotes uniform nucleation of mixed Ni-Co hydroxide intermediates on the NF surface via Ni^2+^/Co^2+^ + 2OH^−^ → Ni(OH)_2_ + Co(OH)_2_. Moreover, the addition of NH_4_F plays a critical role by selectively adsorbing F^−^ ions on high-energy crystal facets, thereby modulating surface energy, suppressing uncontrolled particle aggregation, and directing anisotropic growth into interconnected ultrathin nanosheets. Upon annealing these hydroxide intermediates undergo dehydration and oxidation (Ni(OH)_2_ + 2Co(OH)_2_ + O_2_ → NiCo_2_O_4_ + 3H_2_O), leading to a crystalline spinel NiCo_2_O_4_ phase while preserving the porous, interconnected architecture. Whereas the decomposition urea generates OH^−^ ions in a controlled yet relatively slower manner through the reaction CO(NH_2_)_2_ + 3H_2_O → 2NH_4_^+^ + CO_3_^2−^ + 2OH^−^. The simultaneous evolution of gaseous species and the presence of CO_3_^2−^ anions induce lattice distortion and inter-sheet spacing. This slower reaction kinetics reduces instantaneous nucleation density and favors oriented crystal growth, leading to compact and stacked nanosheets layers. Therefore, hydrolysis behavior of the base source critically determines nucleation dynamics, growth rate, and mass transport during hydrothermal synthesis, ultimately dictating nanosheet assembly.

### 3.3. Material Characterization

The surface elemental composition and chemical states were further probed by XPS and the spectrum was measured using a ULVAC-PHI VersaProbe scanning microprobe instrument (PHI 5000, Chigasaki, Japan). The narrow-ranged spectra were further recorded for all constituent elements, with binding energies referenced to the adventitious carbon C 1s signal. Morphological evolution and surface architecture were explored by FE-SEM using JEOL instrument (JSM-6701F, Tokyo, Japan). Elemental identification and distribution across the electrode surface were obtained via EDS coupled to the FE-SEM. Structural verification of the NiCo_2_O_4_ nanosheets were carried out by X-ray diffraction using a Rigaku SmartLab diffractometer (Akishima, Japan) equipped with Cu Kα radiation (λ = 1.5406 Å). Measurements were performed at ambient conditions within a specified 2θ spectral window of 20–80° and the instrument was operated at 40 kV.

### 3.4. Catalytic HER Test of NCO-U and NCO-HT Electrodes

The electrochemical OER performances of the NCO-H and NCO-U catalyst electrodes were evaluated using a standard three-electrode configuration on a computer-controlled electrochemical workstation. The NCO-H and NCO-U served directly as the working electrode, while a platinum wire and a SCE were employed as the counter and reference electrodes, respectively. All measurements were carried out in 1.0 M KOH aqueous electrolyte at room temperature. The LSV curves were recorded at a slow scan rate to assess OER activity. The Nyquist impedance (EIS) curve measurements were performed at the OER operating potential over a wide frequency range (0.01–10 kHz) to investigate charge-transfer resistance and interfacial kinetics. The *ECSA* were estimated from non-Faradaic cyclic voltammetry measurements conducted within a potential window where no redox processes occur and the double-layer capacitance (*C*_NFC_) values were obtained by analyzing the linear relationship between scan rate and capacitive current density.

## 4. Conclusions

In this work, we have demonstrated a precursor-controlled strategy to regulate the structural, surface, and electrochemical properties of spinel NiCo_2_O_4_ catalysts for alkaline OER. By employing hexamethylenetetramine and urea as distinct agents, two architecturally different NiCo_2_O_4_ catalyst electrodes (NCO-H and NCO-U) were directly grown on conductive nickel foam. Structural analyses confirmed the formation of a pure cubic spinel phase, while electron microscopy revealed that the variation in the precursor promotes the development of ultrathin, highly interconnected two-dimensional nanosheet networks (NCO-H), in contrast to the comparatively compact lamellar morphology (NCO-U). The electrochemical OER evaluation in 1.0 M KOH demonstrated that the interconnected 2D NCO-H nanosheet catalyst delivers superior OER activity across a wide current density range, exhibiting lower overpotentials of 259 mV at 10 mA cm^−2^, a smaller Tafel slope of 84 mV dec^−1^, and faster reaction kinetics (TOF) than NCO-U catalyst (305 mV and 100 mV dec^−1^). The improved performance is attributed to the synergistic effects of the open nanosheet architecture, enhanced electrolyte accessibility, and reduced charge-transfer resistance, which collectively facilitate efficient adsorption and transformation of oxygen intermediates. Further, the long-term chronopotentiometric testing further confirmed the excellent operational stability of NCO-H, with only a minor initial potential adjustment associated with surface reconstruction into an oxyhydroxide-rich active phase, followed by highly stable OER operation for extended durations up to 50 h. The findings provide valuable insights into rational morphology engineering of spinel oxides and offer a scalable and effective pathway toward designing high-performance, durable, and earth-abundant OER electrocatalysts for alkaline water electrolysis.

## Data Availability

The data related to this article will be made available by the corresponding authors on reasonable request.

## Supplementary Materials

The following supporting information can be downloaded at: https://www.mdpi.com/article/10.3390/ijms27031444/s1, [47,48,49,50,51,52,53,54,55,56].

## Abbreviations

The following abbreviations are used in this manuscript:

FE-SEM	Field-emission scanning electron microscopy
*A*	Geometric area of the electrode
TOF	Turnover frequency
HER	Hydrogen evolution reaction
*F*	Faraday’s constant
*n*	Number of electrons transferred per oxygen molecule
XRD	X-ray diffraction
RHE	Reversible hydrogen electrode
*ECSA*	electrochemically active surface area
CV	Cyclic voltammetry
SCE	Saturated calomel electrode
EIS	Electrochemical impedance spectroscopy
*η*	Overpotential
EDX	Energy-dispersive X-ray spectroscopy
*R*ct	Charge-transfer resistance
XPS	X-ray photoelectron spectroscopy
*C*_NFC_	Non-faradaic capacitance
