# Supplementary material for: Morphology-Driven Enhancement of Alkaline OER Performance in Spinel NiCo2O4 Nanosheet Electrodes
Source: Int J Mol Sci. 2026 Jan 31;27(3):1444. doi: 10.3390/ijms27031444 (PMC12898273; doi:10.3390/ijms27031444)
Supplement: Supplementary file 1 [file ijms-27-01444-s001.zip › ijms-4127384-supplementary.pdf]

Supporting Information

Morphology-Driven Enhancement of Alkaline OER Performance in Spinel $NiCo_2O_4$ Nanosheet Electrodes

Abu Talha Aqueel Ahmed [1], Abu Saad Ansari [2], Sangeun Cho [1] and Atanu Jana [1,*]

[1] Division of System Semiconductor Science, Dongguk University, Seoul 04620, Republic of Korea

[2] Nano Center Indonesia Research Institute, Puspiptek Street, South Tangerang, Banten 15314, Indonesia

Corresponding Author: atanujana@dongguk.edu

Supporting Figures

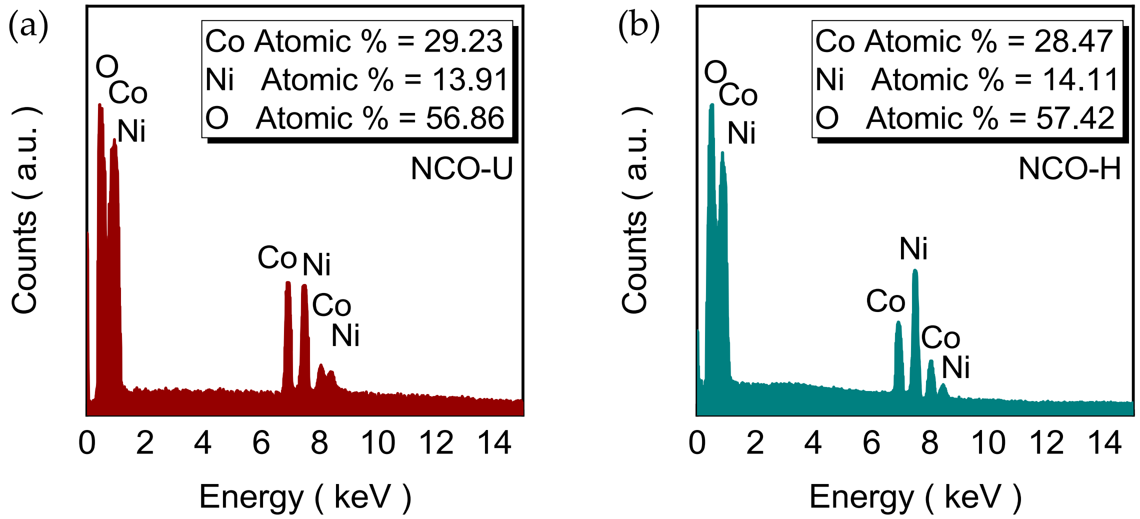

Figure S1. EDS spectra of (**a**) NCO-H and (**b**) CNO-U catalyst electrodes with inset tables summarizing the atomic percentage of the detected constituent elements.

Table S1. Comparative electrocatalytic OER activities of the optimized $NiCo_2O_4$ catalyst and recently reported Ni-/Co-oxide-based catalysts evaluated in an alkaline 1.0 M KOH medium.

No.	Catalyst electrodes	Overpotential (mV) @10 (mA cm^{-2})	Tafel slope (mV dec^{-1})	Stability at *J* (*J* in mA cm^{-2})	Supporting Reference
1	Ni-Fe-OH@Ni_3S_2/NF	165	93	50 h@100	[47]
2	CoS/MoS_2 HS	294	31	100 h@100	[48]
3	NiFe-NF	290	50.1	30 h@100	[49]
4	$NiCo_2O_4$	314	116	-	[50]
5	NCOC	228	108	100 h@15	
6	CCO-300 nanosheets	331	48	10@100	[51]
7	$NiCo_2O_4$/NiO	360	61	11@1.64 V	[52]
8	$Ni_{0.75}Cu_{0.25}Co_2O_4$/GF	509	119	10@10	[53]
9	N-CoS_2	271	63	75@500	[54]
10	$NCP_{0.5}$@C_3N_4	247	48	24@10	[55]
11	NCO	440	93	-	
12	$Ni_{50}Fe_{50}$-DAT	300	–	72 h@100	[56]
**13**	**NCO-H**	**259**	**84**	**100 h@10**<br>**100 h@250**	**Present work**

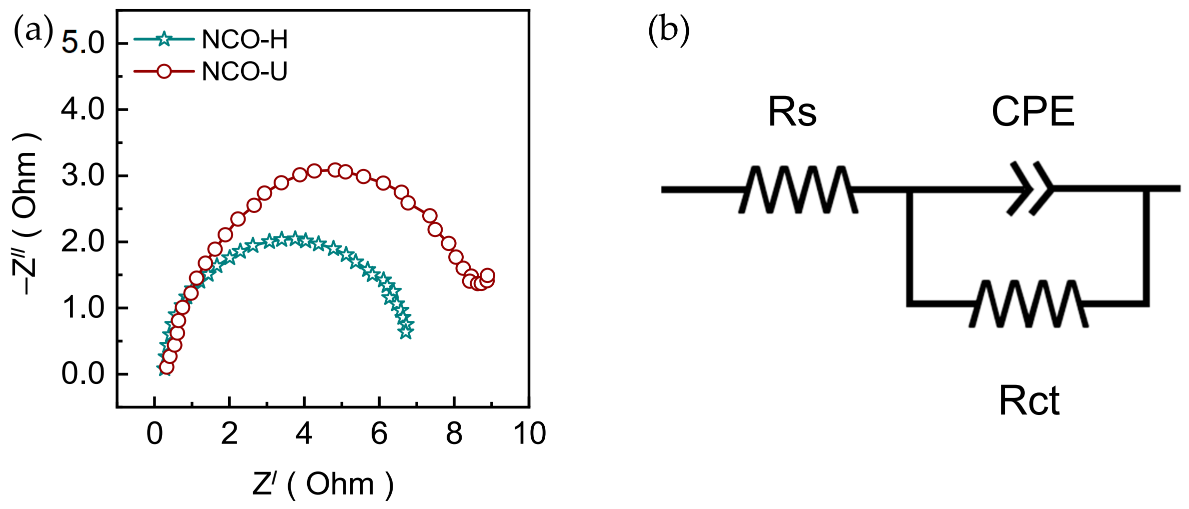

Figure S2. (**a**) Nyquist impedance curves of the NCO-H and NCO-U catalysts measured under identical conditions in an alkaline KOH medium and (**b**) Equivalent tank circuit used to fit the both EIS curves.

Table S2. Equivalent-circuit fitting parameters derived from Nyquist impedance spectra of NCO-H and NCO-H catalysts using Z-View software.

Catalysts	Before stability		After OER stability	
	R_S (Ω)	R_{ct} (Ω)	R_S (Ω)	R_{ct} (Ω)
**NCO-H**	0.273	6.91	0.276	6.93
**NCO-U**	0.325	8.84	-	-

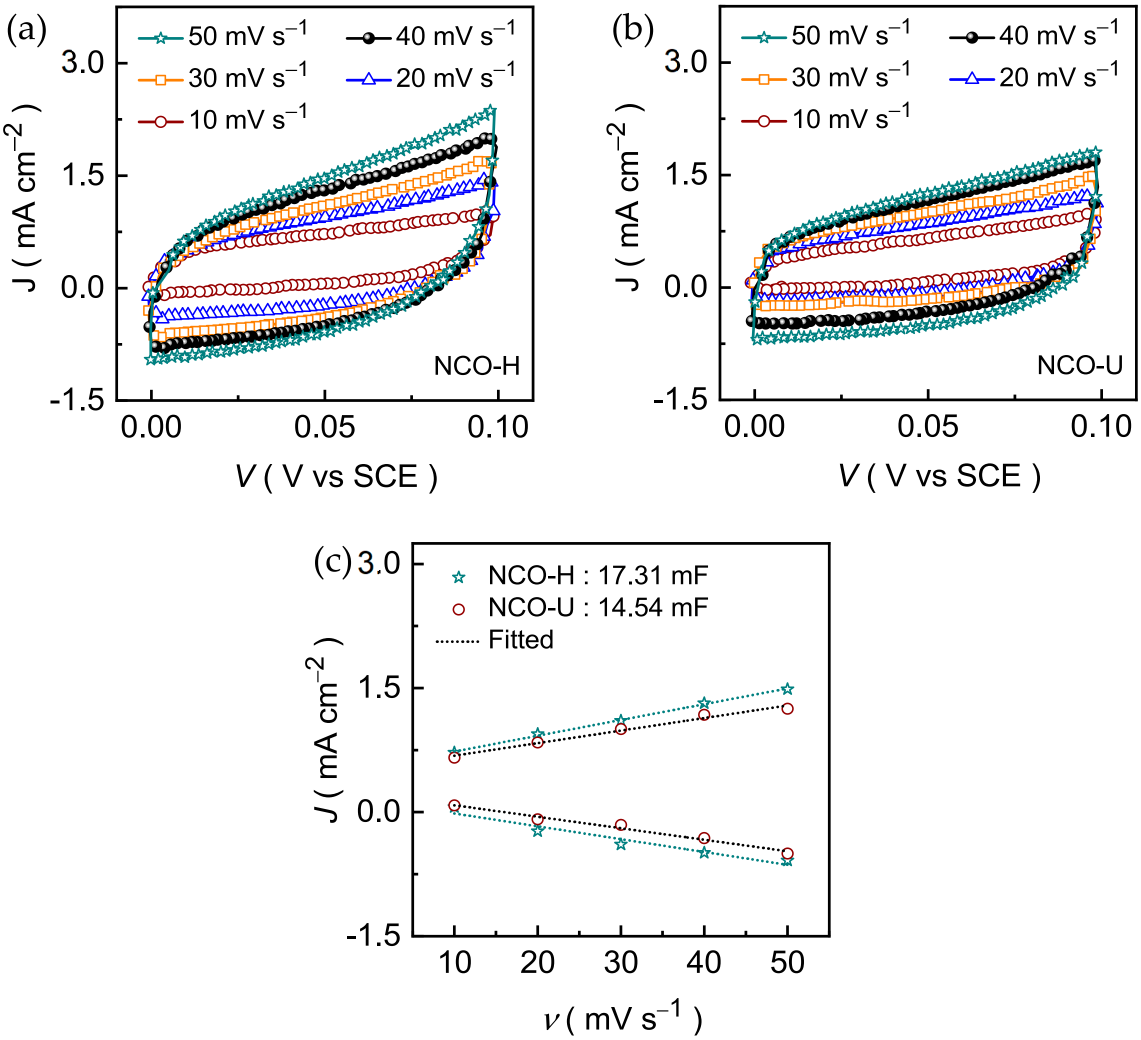

Figure S3. Non-Faradaic CV curves recorded at different scan rates for (**a**) NCO-H and (**b**) NCO-U catalysts. (**c**) The non-Faradaic current density extracted at 0.05 V (vs. SCE) as a function of scan rate was used to estimate the double layer capacitance (C_{DL}) and accessible electrocatalytically active sites in terms of *ECSA* for NCO-H and NCO-U catalysts.

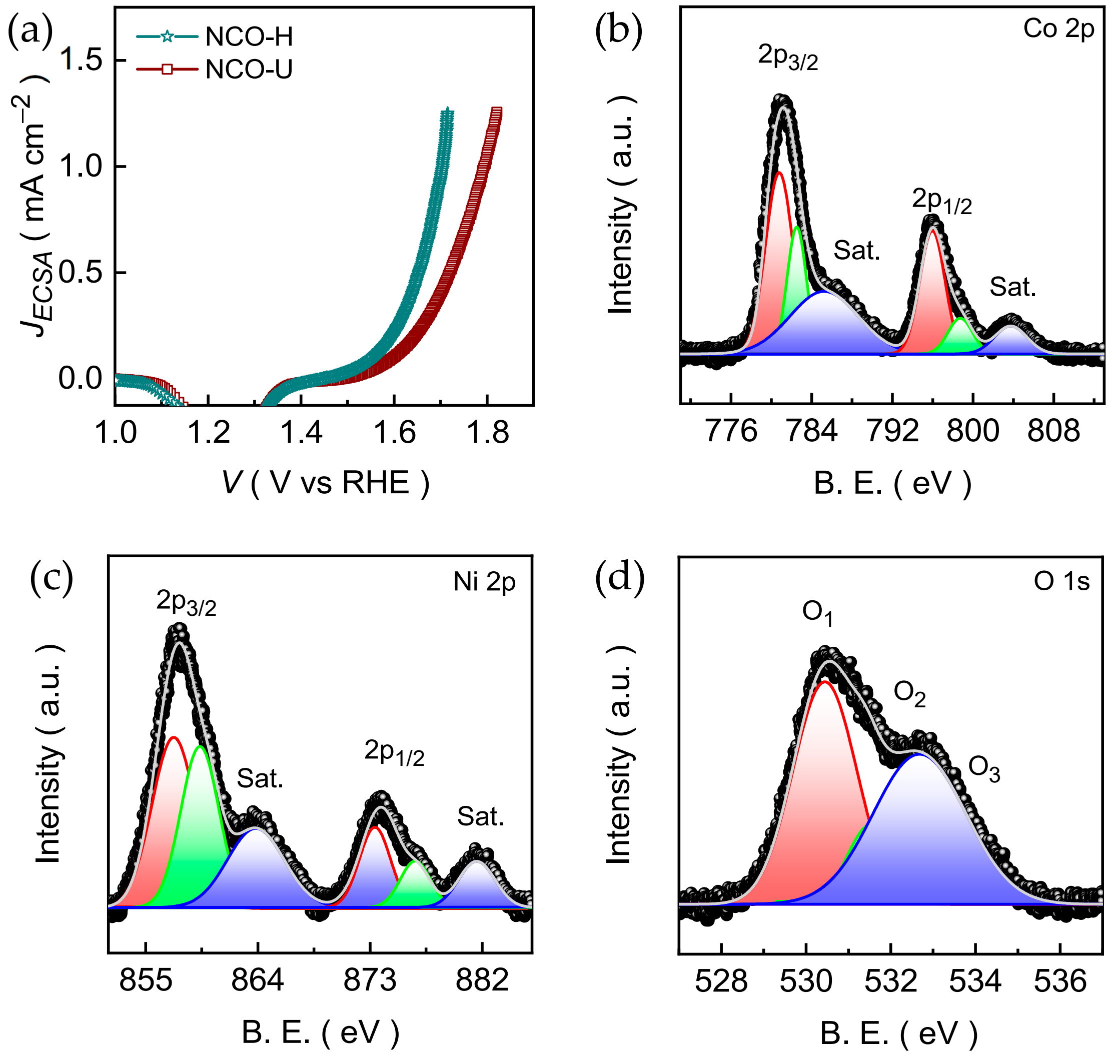

Figure S4. (**a**) ECSA-corrected LSV curves for NCO-H and NCO-U catalysts. Post-stability measured high-resolution (**b**) Co 2p, (**c**) Ni 2p, and (**d**) O 1s XPS emission spectrum for the NCO-H catalyst.

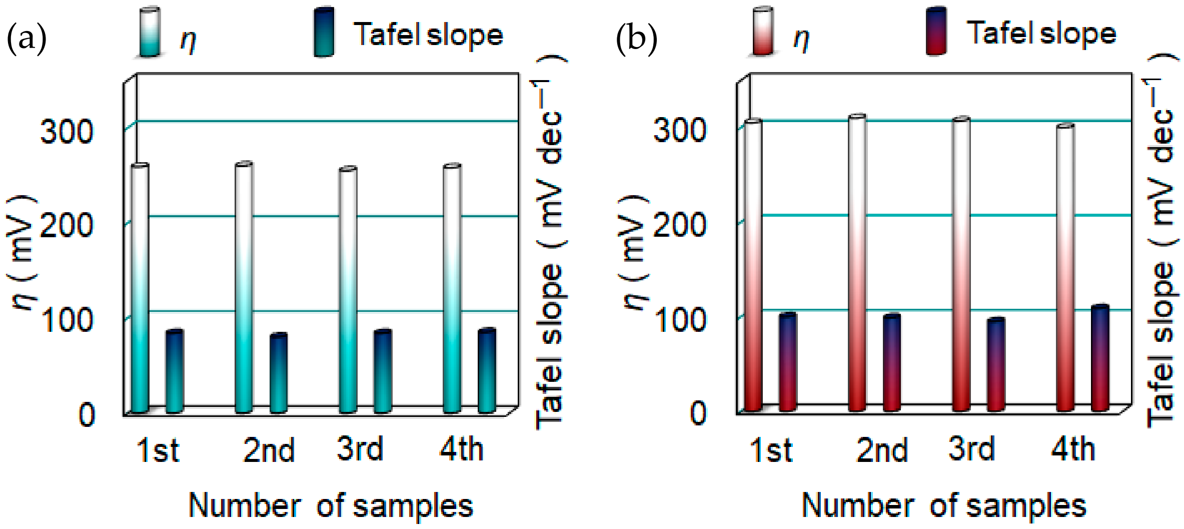

Figure S5. Reproducibility evaluation of (**a**) NCO-H and (**b**) NCO-U catalysts evaluated using independently fabricated electrodes tested under the same experimental conditions.

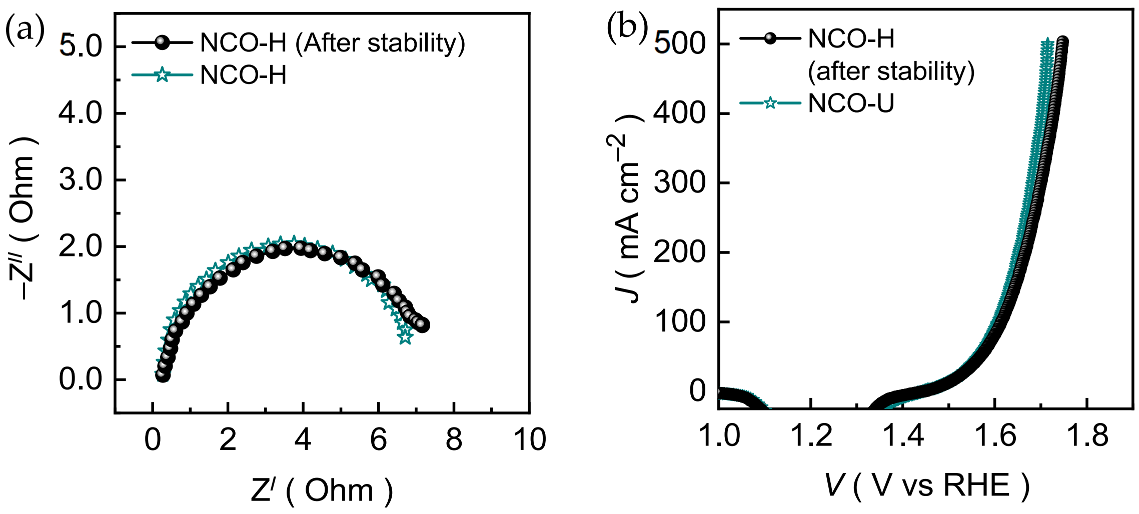

Figure S6. Post-stability measured (**a**) Nyquist impedance curves and (**b**) polarization curves of the NCO-H catalyst.

Supporting References

47. Zou, X.; Liu, Y.; Li, G.-D.; Wu, Y.; Liu, D.-P.; Li, W.; Li, H.-W.; Wang, D.; Zhang, Y.; Zou, X., Ultrafast Formation of Amorphous Bimetallic Hydroxide Films on 3D Conductive Sulfide Nanoarrays for Large-Current-Density Oxygen Evolution Electrocatalysis. *Adv. Mater.* **2017,** *29* (22), 1700404.
48. Talha Aqueel Ahmed, A.; Ho Lee, C.; Saad Ansari, A.; Pawar, S. M.; Han, J.; Park, S.; Shin, G.; Yeon, S.; Cho, S.; Seol, J.; Uck Lee, S.; Kim, H.; Im, H., Hybridized heterostructure of CoS and MoS_2 nanoparticles for highly-efficient and robust bifunctional water electrolysis. *Appl. Surf. Sci.* **2022,** *592*, 153196.
49. Guo, D.; Qi, J.; Zhang, W.; Cao, R., Surface Electrochemical Modification of a Nickel Substrate to Prepare a NiFe-based Electrode for Water Oxidation. *ChemSusChem* **2017,** *10* (2), 394-400.

50. Xu, S.; Zhang, P.; Zhao, R.; Wook Bae, J.; Li, H.; Yong Lee, J.; Yoo, P. J., Engineered oxidation states in $NiCo_2O_4$@CeO_2 nanourchin architectures with abundant oxygen vacancies for enhanced oxygen evolution reaction performance. *Chem. Eng. J.* **2024,** *482*, 148787.

51. Ahmed, A. T. A.; Mujtaba, M. M.; Ansari, A. S.; Cho, S., Binder-Free Spinel Co_2CuO_4 Nanosheet Electrodes with Cu-Driven Kinetic Enhancement for Alkaline OER Applications. *Materials* **2026,** *19* (2), 301.

52. Mahala, C.; Basu, M., Nanosheets of NiCo2O4/NiO as Efficient and Stable Electrocatalyst for Oxygen Evolution Reaction. *ACS Omega* **2017,** *2* (11), 7559-7567.

53. Park, H.; Park, B. H.; Choi, J.; Kim, S.; Kim, T.; Youn, Y.-S.; Son, N.; Kim, J. H.; Kang, M., Enhanced Electrochemical Properties and OER Performances by Cu Substitution in $NiCo_2O_4$ Spinel Structure. *Nanomaterials* **2020,** *10* (9), 1727.

54. Ahmed, A. T. A.; Ansari, A. S.; Nugroho, F. G.; Kim, J.; Im, H.; Cho, S., Electronic Structure Tuning of CoS_2 via N-Heteroatom Doping for Efficient Oxygen Evolution Reaction Application. *Int. J. Energy Res.* **2025,** *2025* (1), 4507049.

55. Li, S.-F.; Li, X.; Yan, D., $NiCo_2O_4$ Electrocatalyst Doped with Phosphorus for Improved Oxygen Evolution Reaction. *ACS Appl. Nano Mater.* **2024,** *7* (11), 13358-13366.

56. Hoang, T. T. H.; Gewirth, A. A., High Activity Oxygen Evolution Reaction Catalysts from Additive-Controlled Electrodeposited Ni and NiFe Films. *ACS Catal.* **2016,** *6* (2), 1159-1164.
